# Disruption of the c‐terminal serine protease domain of *Fam111a* does not alter calcium homeostasis in mice

**DOI:** 10.14814/phy2.15977

**Published:** 2024-05-02

**Authors:** Rebecca Siu Ga Tan, Christy Hui Lin Lee, Wanling Pan, Serene Wohlgemuth, Michael R. Doschak, R. Todd Alexander

**Affiliations:** ^1^ Department of Physiology University of Alberta Edmonton Alberta Canada; ^2^ Membrane Protein Disease Research Group University of Alberta Edmonton Alberta Canada; ^3^ The Women and Children's Health Research Institute Edmonton Alberta Canada; ^4^ Department of Medicine University of Alberta Edmonton Alberta Canada; ^5^ Faculty of Pharmacy & Pharmaceutical Sciences University of Alberta Edmonton Alberta Canada; ^6^ Department of Pediatrics University of Alberta Edmonton Alberta Canada

**Keywords:** calcium, FAM111A, Kenney–Caffey syndrome, Osteocraniostenosis

## Abstract

*FAM111A* gene mutations cause Kenney–Caffey syndrome (KCS) and Osteocraniostenosis (OCS), conditions characterized by short stature, low serum ionized calcium (Ca^2+^), low parathyroid hormone (PTH), and bony abnormalities. The molecular mechanism mediating this phenotype is unknown. The c‐terminal domain of FAM111A harbors all the known disease‐causing variations and encodes a domain with high homology to serine proteases. However, whether this serine protease domain contributes to the maintenance of Ca^2+^ homeostasis is not known. We hypothesized the disruption of the serine protease domain of FAM111A would disrupt Ca^2+^ homeostasis. To test this hypothesis, we generated with CRISPR/Cas9, mice with a frameshift insertion (c.1450insA) or large deletion (c.1253‐1464del) mutation in the *Fam111a* serine protease domain. Serum‐ionized Ca^2+^ and PTH levels were not significantly different between wild type, heterozygous, or homozygous *Fam111a* mutant mice. Additionally, there were no significant differences in fecal or urine Ca^2+^ excretion, intestinal Ca^2+^ absorption or overall Ca^2+^ balance. Only female homozygous (c.1450insA), but not heterozygous mice displayed differences in bone microarchitecture and mineral density compared to wild‐type animals. We conclude that frameshift mutations that disrupt the c‐terminal serine protease domain do not induce a KCS or OCS phenotype in mice nor alter Ca^2+^ homeostasis.

## 1 INTRODUCTION

Regulation of Ca^2+^ homeostasis is dependent on the parathyroid glands which help to coordinate actions of the kidney, intestine, and bone (Sadideen & Swaminathan, 2004; Sato et al., 2017; Tan et al., 2021). Lower plasma Ca^2+^ levels result in parathyroid hormone (PTH) release from the parathyroid glands. PTH reduces Ca^2+^ excretion in the urine and stimulates the synthesis of calcitriol which increases intestinal Ca^2+^ absorption. PTH also increases Ca^2+^ release from the bone. Kenney–Caffey syndrome type II (KCS, OMIM #127000) and Osteocraniostenosis (OCS, OMIM #602361) are rare genetic conditions characterized by disrupted Ca^2+^ homeostasis. These patients display hypocalcemia, inappropriately low PTH levels, and abnormalities of bone, eyes, and head (Rosato et al., 2022; Schigt et al., 2023; Unger et al., 2013). Individuals with the non‐lethal, autosomal dominant, KCS have a phenotype that includes cortical thickening and medullary stenosis of tubular bones, short stature, microphthalmia, delayed closure of the anterior fontanel, frontal bossing, and triangular faces (Schigt et al., 2023; Unger et al., 2013). Intellectual disability and chronic kidney disease have also been reported (Schigt et al., 2023). Characteristics of the perinatally lethal OCS include thin and gracile bones, cloverleaf‐shaped skulls, and microphthalmia (Rosato et al., 2022; Unger et al., 2013).

Heterozygous mutations in the *FAM111A* (Family with sequence similarity 111 member A) gene cause KCS and OCS, although the underlying molecular mechanisms mediating the clinical phenotype are unknown (Unger et al., 2013). However, global deletion of Fam111a in mice does not cause a similar phenotype (Ilenwabor et al., 2022). Interestingly, all the disease‐causing mutations in FAM111A are in the c‐terminal, trypsin‐like serine protease domain (Unger et al., 2013). This domain contains a catalytic triad (H385‐D439‐S541) which is a characteristic of all serine proteases (Hoffmann et al., 2020; Polgár, 2005). FAM111A displays serine protease activity as evidenced by in vitro work where over‐expression in a cell model resulted in auto‐cleavage products, a process inhibited by the serine protease inhibitor, AEBSF, or when the catalytic site was mutated (D439N) (Hoffmann et al., 2020). FAM111A is also a chromatin‐associated protein that plays a role in DNA replication (Alabert et al., 2014; Hoffmann et al., 2020; Kojima et al., 2020). It interacts with proliferating cell nuclear antigen (PCNA) through its PCNA‐interacting peptide (PIP) box, located in the N‐terminus, which aids in PCNA loading onto chromatin (Alabert et al., 2014). Further, by studying *FAM111A* knockout cells, FAM111A was observed to remove protein obstacles from replication forks, thereby preventing stalling during DNA replication (Kojima et al., 2020). FAM111A protease activity also inhibits DNA replication by displacement of the main PCNA loader, the replication factor C (RFC) complex, from chromatin (Hoffmann et al., 2020; Kang et al., 2019). Finally, FAM111A acts as a host range restriction factor, which the polyomavirus, simian virus 40 (SV40) specifically targets to overcome viral replication restriction (Fine et al., 2012).

The functions of FAM111A identified thus far do not readily explain the role of this gene in the regulation of Ca^2+^ homeostasis. It is therefore perplexing as to why mutations in *FAM111A* cause the severe phenotypes of KCS and OCS in humans. The c‐terminal serine protease domain harbors all the known mutations, implicating its role in disease. Our objective was therefore to generate *Fam111a* mutant mouse models with a disrupted serine protease domain and characterize their phenotype. We hypothesized that disruption of the serine protease domain of *Fam111a* would negatively impact Ca^2+^ homeostasis in mice. When we started our animal experiments in the year 2020, there were no reports examining the effects of *Fam111a* mutations in mice. However, a study was published in 2022, which found that *Fam111a* knockout in a C57BL/6 mouse model had no adverse effects in the mice (Ilenwabor et al., 2022). They used a ZEN‐UB1 cassette to delete the entire protein coding region of *Fam111a*. In our study, we use CRISPR‐Cas9 gene‐editing technology to introduce a frameshift insertion or deletion mutation in the c‐terminus, serine protease domain of *Fam111a* in FVB/N mice.

## 2 MATERIALS AND METHODS

### 2.1 Animal ethics

All experimental procedures were approved by the University of Alberta's Animal Care and Use Committee for Health Sciences (AUP00000213).

### 2.2 Generation of Fam111a mutant mice

**FIGURE 1:**
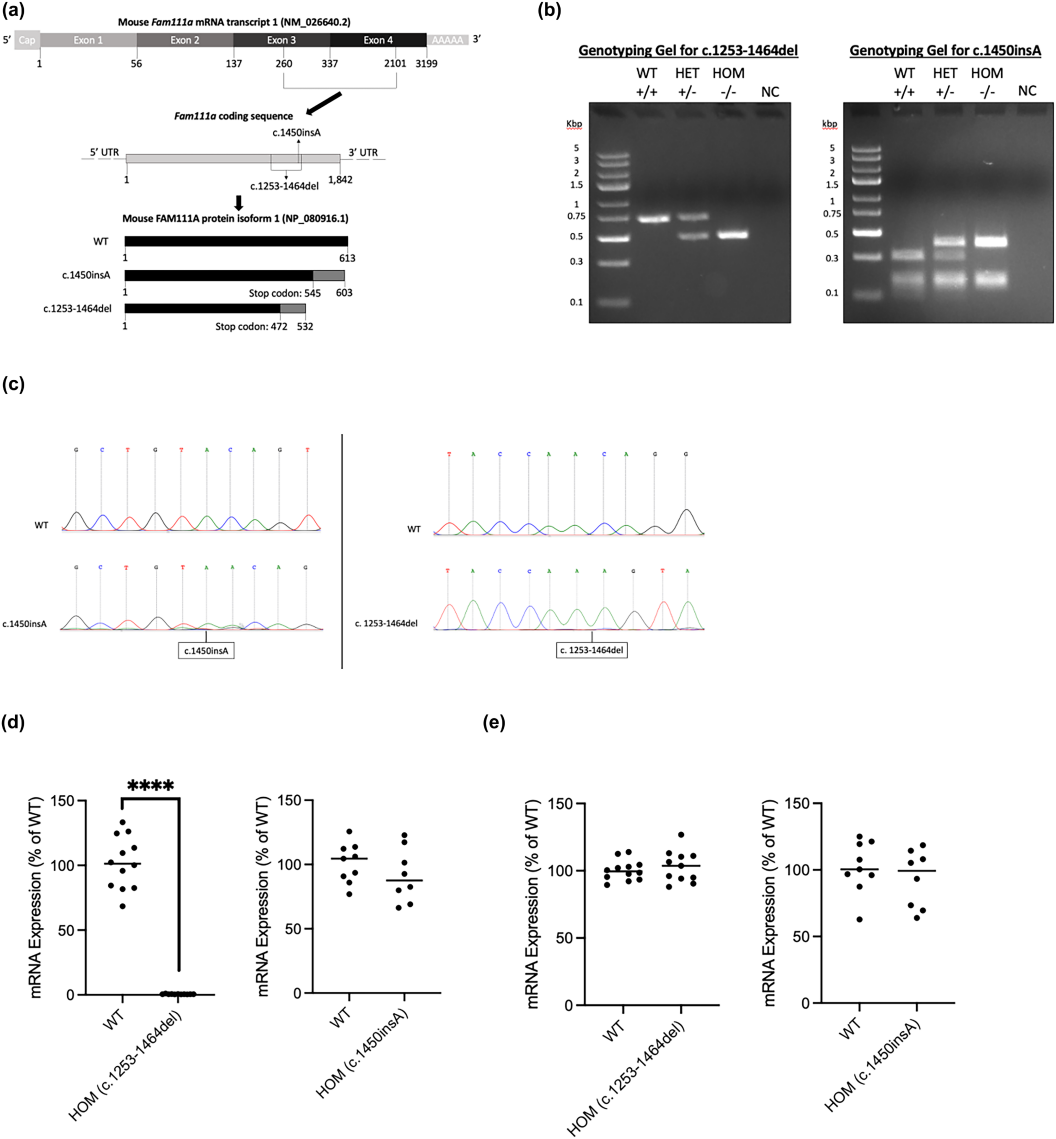
*Fam111a* mutant mouse model. (a) Schematic of the 3199 bp mouse *Fam111a* mRNA transcript 1 (NM_026640.2) which contains four exons. The 1842 bp coding sequence of *fam111a* is located within exons three and four corresponding to 260–2101 bp of the transcript. The c.1450insA and c.1253‐1464del mutations are also depicted. WT, c.1450insA, and c.1253‐1464del proteins are 613, 603, and 532 amino acids respectively. The c.1450insA, and c.1253‐1464del proteins contain early stop codons at amino acid 545 and 472, respectively. (b) Genotyping gels for *Fam111a* mutants showing WT (+/+), HET (+/−), HOM (−/−), and negative control (NC). (c) Sanger sequencing electropherograms of *Fam111a* WT and mutants. (d and e) Relative *Fam111a* mRNA expression in the kidneys of WT (*N* = 12 or 9), HOM (c.1253‐1464del, *N* = 11), and HOM (c.1450insA, *N* = 8) mice when using primers targeting the mutated, c‐terminal region of *Fam111a* (d) or primers targeting the non‐mutated, n‐terminal region of *Fam111a* (e). Expression is normalized to 18S rRNA housekeeping gene and then normalized to the average WT expression. Data are presented as mean ± SD. The Student's *t*‐test was used to compare means and significance is denoted by an asterisk (**** *p* < 0.0001).

Gene editing was performed on FVB/N mice using CRISPR‐Cas9 technology (Jiang & Doudna, 2017; Wang et al., 2013) to generate strains that have a *Fam111a* mutation at the University of Alberta's Transgenic Core Facility. Fam111a‐specific ribonucleoprotein complexes were formed by combining equimolar amounts of Alt‐R CRISPR‐Cas9 tracrRNA (Integrated DNA Technologies [IDT], 1072532) and Fam111a crispr RNA, heating to 95°C for 10 min and cooling to room temperature before adding Alt‐R S.p. Cas9 nuclease V3 (IDT, 1081058). Newly fertilized FVB/N zygotes were washed into 10 μL Opti‐MEM (Gibco, 31985062) and mixed with the RNP complexes in 10 μL Opti‐MEM. The embryos and RNP complexes were transferred to a 0.1 cm electroporation cuvette (Bio‐Rad, 1652083) and electroporation was performed in a Bio‐Rad GenePulser XCell using three cycles of 3.0 ms at 30 V with a 100 ms interval between pulses. The zygotes were cultured overnight in KSOM media (Millipore, MR‐121‐D) in a MINC benchtop incubator (Cook Medical; 37°C, 5% CO_2_, and 5% O_2_/nitrogen) and viable embryos were surgically transferred to pseudopregnant CD1 female mice the following day. The resultant pups were genotyped for the novel insertions or deletions (indel), created via non‐homologous end joining, in the *Fam111a* gene using Sanger sequencing. Polymerase chain reaction (PCR) of mouse ear notch DNA was used to confirm genotypes of progeny (primers used were Forward: CTAGCATTGTGGGTGAAGGC; Reverse: GACTAAACCCTTTCCAGCCCTC). The PCR program was as follows: 98°C for 3 min followed by 29 cycles of; 98°C for 5 s, 64°C for 5 s, 72°C for 20 s, and then finally 72°C for 1 min. The wild‐type reaction produced a band at 706 bp, the heterozygous deletion mutant reaction resulted in two bands (706 and 495 bp), and the homozygous deletion reaction produced a band at 495 bp (Figure 1b). A digestion step after the PCR was required to detect the insertion mutants with the restriction enzyme, BsrGI‐HF (New England Biolabs), in CutSmart buffer (New England Biolabs), at 37°C for 20 min. BsrGI‐HF causes three cuts in the wild‐type DNA product, resulting in four fragments (290, 92, 167, 136 bp). After gel electrophoresis, only two bands (290 and 167 bp) from the wild‐type product were easily observed as the shorter 92 and 136 bp bands were difficult to detect (Figure 1b). BsrGI‐HF does not recognize one of the cut sites in the c.1450insA mutant. Therefore, BsrGI‐HF makes two cuts in the c.1450insA mutant DNA resulting in three fragments (382, 167, 136 bp). After gel electrophoresis, heterozygous insertion mutants readily revealed three bands (382, 290, and 167 bp) with the 136 bp band not usually being visible (Figure 1b). The heterozygous mice display the 290 bp band characteristic of wild‐type reaction while the homozygous insertion mutants readily displayed two bands (382 and 167 bp) with the 136 bp band not generally being visible (Figure 1b). Sanger sequencing was performed on the DNA of WT and mutants by the Molecular Biology and Service Unit in the Department of Biological Sciences at the University of Alberta. The WT and mutant sequences were aligned using NCBI Nucleotide BLAST to identify the specific mutation and location of the mutation (Figure 1c).

### 2.3 Kidney RNA extraction and quantitative real‐time PCR (qPCR)

Kidneys were snap‐frozen in liquid nitrogen after isolation and stored at −80°C for future RNA extraction. RNA was extracted from kidneys using TRIzol (Invitrogen) and processed as previously (Plain et al., 2020). In brief, after DNAse treatment (Invitrogen), cDNA synthesis was performed using the SensiFAST cDNA Synthesis Kit (Bioline). Then, qPCR was used to measure the relative *Fam111a* mRNA expression in the kidneys of WT, HOM (c.1253‐1464del), and HOM (c.1450insA) mice. Relative expression was calculated using the comparative CT method (2^−ΔΔCt^). *Fam111a* expression was first normalized to *18S rRNA* housekeeping gene expression and then normalized to the average WT expression. Quantitative real‐time PCR was performed in triplicate for each sample on the QuantStudio 6 Pro Real‐Time PCR System (Applied Biosystems) using TaqMan Master Mix (Applied Biosystems) and specific primers and probes that were designed using the PrimerQuest Tool (Integrated DNA Technologies). We designed primers targeting the mutated, c‐terminal region of *Fam111a* (Figure 1d; Forward: TGCCACATAGTGGGTTGATTTA; Reverse: CTGCTTCCTCTCTTGCTTGA; Probe: ACAGTGGTCCCTCAAAGTAGTAGAAGA) or primers targeting the non‐mutated, n‐terminal region of *Fam111a* (Figure 1e; Forward: CTCAGTTGGGTTCCTATGGTG; Reverse: TCTATGCCTTCACCCACAATG; Probe: ACAGCCTGCATTTCCATTGTTGTTCC). The *18S rRNA* housekeeping primers (assay ID Mm03928990_g1, catalog no. 4351370) were obtained from Life Technologies (Applied Biosystems). The amplification program consisted of preincubation at 50°C for 2 min and 95°C for 10 min followed by 40 cycles of 95°C for 15 s and 60°C for 1 min.

### 2.4 Metabolic cage studies

Prior to placement into the metabolic cage, mice were housed in static cages with regular light/dark cycles. Metabolic cage studies were performed as previously reported (Beggs et al., 2021; Dimke et al., 2013; Plain et al., 2020). Heterozygous (HET), homozygous (HOM), and wild type (WT) mice (4–5 months old, both sexes) were placed into metabolic cages for 3 days. The total number of animals used was 62. For the metabolic cage experiments involving the c.1253‐1464del mutant, we used the following number of animals: WT (female *N* = 6, male *N* = 6), HET (female *N* = 6, male *N* = 6), and HOM (female *N* = 6, Male *N* = 5). For the experiments using c.1450insA mice, we used the following number of animals: WT (female *N* = 5, male *N* = 4), HET (female *N* = 4, male *N* = 6), and HOM (female *N* = 5, male *N* = 3). All mice survived the experiments until euthanasia, which was performed by first anesthetizing mice with isoflurane and then euthanasia by cervical dislocation. Mice received a standard diet (PicoLab® Rodent Diet 5053) with ad libitum access to food and water. Every 24 h mice were weighed, and the amount of chow and water consumed was measured. Feces and urine from each mouse were collected daily, but only the samples obtained on day three were used for analysis. After the third 24‐h collection, blood was obtained by cardiac puncture and electrolytes including ionized Ca^2+^, glucose, blood urea nitrogen (BUN), hematocrit (Hct) and hemoglobin (Hb) were measured using a blood gas handheld analyzer (Vet Scan i‐STAT 1 Analyzer, Abaxis) with a CHEM8+ cartridge (Abbott Laboratories). Mice were euthanized and tissue including kidneys, intestine, and bone collected as previously (Beggs et al., 2021).

### 2.5 Measurement of urine, fecal, and serum ion concentration, serum and urine creatinine, and serum PTH

Urine and serum creatinine were measured using the Creatinine Parameter Assay Kit (R&D Systems) and the Diazyme Creatinine Assay Kit (Diazyme Laboratories), respectively. The absorbance from the colorimetric assay was measured using a Synergy MX plate reader and Gen5 software (Agilent BioTek). Feces were dried in an incubator (Imperial III Incubator, Labline, Mumbai, India) at 65°C for 72 h after collection. As previously described (Beggs et al., 2021; Ferreira et al., 2021; Plain et al., 2020), feces were then homogenized using a mortar and pestle. Then, 1 mL of 0.6 M hydrochloric acid was added to 50 mg of ground feces. The mixture was rotated for 72 h and then the supernatant was collected after centrifugation at 2000*g* for 5 min at 4°C. The concentration of ions in the urine and feces was determined using a Dionex Aquion Ion Chromatography (IC) System and Chromeleon 7 Chromatography Data System software (Thermo Fisher Scientific Inc.) as previously (Beggs et al., 2021; Plain et al., 2020). Serum PTH was measured using the mouse PTH 1–84 ELISA Kit (Immutopics Inc.) according to the manufacturer's instructions.

The fractional excretion of ions (X) was calculated as:

$$\mathrm{FEx}=\frac{\left[\text{Urine}\;X\right]*\left[\text{Serum Creatinine}\right]\;}{\left[\text{Urine Creatinine}\right]*\left[\text{Serum}\;X\right]}*100\%$$

The urinary ion to creatinine ratio was calculated as [Urine Ion]/[Urine Creatinine].

The Ca^2+^ bioavailability and balance were calculated as follows:

$$\begin{array}{c} {\mathrm{Ca}}^{2+}\text{bioavailability}=\frac{\mathrm{mg}\;\text{of}\;{\mathrm{Ca}}^{2+}\;\text{ingested}–\mathrm{mg}\;\text{of}\;{\mathrm{Ca}}^{2+}\;\text{in feces}}{\mathrm{mg}\;\text{of}\;{\mathrm{Ca}}^{2+}\;\text{ingested}}*100\% \end{array}$$

$${\mathrm{Ca}}^{2+}\;\text{balance}=\left(\mathrm{mg}\;{\mathrm{Ca}}^{2+}\;\text{ingested}\right)–\left(\mathrm{mg}\;{\mathrm{Ca}}^{2+}\;\text{in feces}\right)–\left(\mathrm{mg}\;{\mathrm{Ca}}^{2+}\;\text{in urine}\right)$$

### 2.6 Measurement of bone mineral density and morphometry

The bone mineral density and morphometry of tibia was measured as previously (Beggs et al., 2021). Leg bones were fixed in 4% paraformaldehyde and stored at 4°C before analysis. The right tibia was scanned using a Skyscan 1176 micro‐computed tomography (micro‐CT) imager (Skyscan NV, Kontich, Belgium) at a resolution of 9 μm with the following settings: Voltage (45 kV), current (555 μA), filter (Al 0.2 mm), rotation step (0.7°), and exposure (750 ms). Micro‐CT images were reconstructed using NRecon software (v.1.6.9.18) and the DataViewer (v.1.5.1.2). The CT‐analyzer (v.1.14.4.1) software was used to analyze 100 slices of the tibial metaphysis starting from 20 slices below the metaphyseal growth plate. CTVol software (v.2.2.3.0) was used for 3D visualization of bone.

### 2.7 Statistical analysis

GraphPad Prism (v.9.4.1) was used for statistical analysis of data. For the qPCR data, the Student's *t*‐test was used to compare means of two groups. Two‐way analysis of variance (ANOVA) was used to identify the effects of sex and genotype on a given variable and multiple comparisons were made using Šidák's test. Adjusted *p*‐values of <0.05 were considered statistically significant.

## 3 RESULTS

We generated mice with either an insertion frameshift mutation (c.1450insA) or a large deletion frameshift mutation (c.1253‐1464del) in the c‐terminal serine protease domain of *Fam111a* (Figure 1). This resulted in the mutant mice having a premature stop codon at 545 and 472 of the protein, respectively (Figure 1a). Using specific quantitative real‐time PCR primers for the serine protease domain, we show that this region of the *Fam111a* mRNA is not detected in the c.1253‐1464del HOM mutants (Figure 1d). However, we still detect the region in the c.1450insA HOM mutants due to the mutation only being a single nucleotide change. When we use primers targeting the non‐mutated, n‐terminus region of *Fam111a*, both mutants express similar mRNA levels of *Fam111a* compared to WT (Figure 1e).

Female and male HET or HOM mice carrying either the *Fam111a* c.1450insA or the c.1253‐1464del mutation appeared similar in appearance and had indistinguishable body weight to WT mice of the same sex (Tables 1 and 2). Additionally, metabolic cage studies revealed that HET and HOM *Fam111a* mutants had indistinguishable water and chow consumption, as well as urine volume, urine pH, and fecal mass (Tables 1 and 2).

**TABLE 1:** Metabolic cage balance results for *Fam111a* c.1253‐1464del mice.

Day 3 (48–72 h) metabolic cage measurement	Sex	WT Mean ± SD	HET Mean ± SD	HOM Mean ± SD	Two‐way ANOVA interaction *p*‐value	Two‐way ANOVA sex *p*‐value	Two‐way ANOVA genotype *p*‐value	Šidák's multiple comparisons (Adjusted *p*‐value <0.05)
Body weight (g)	♀	28.45 ± 3.62	29.74 ± 3.76	30.95 ± 3.55	0.146	0.0003	0.681	♂ WT > ♀ WT ♂ HET > ♀ HET
	♂	34.84 ± 2.84	35.56 ± 3.07	32.33 ± 1.79				
Food intake (g)	♀	4.59 ± 0.90	4.70 ± 0.30	4.28 ± 0.81	0.277	0.445	0.972	NS
	♂	4.36 ± 0.43	4.19 ± 0.38	4.56 ± 0.36				
Water intake (mL)	♀	4.01 ± 0.86	5.05 ± 0.83	4.83 ± 1.16	0.162	0.084	0.279	NS
	♂	4.20 ± 0.48	4.02 ± 0.39	4.28 ± 0.51				
Urine volume (mL)	♀	1.47 ± 0.27	1.72 ± 0.77	1.66 ± 0.34	0.372	0.063	0.983	NS
	♂	2.13 ± 0.56	1.84 ± 0.40	1.86 ± 0.46				
Urine pH	♀	6.10 ± 0.17	6.27 ± 0.30	6.23 ± 0.08	0.597	0.662	0.284	NS
	♂	6.20 ± 0.13	6.23 ± 0.08	6.24 ± 0.09				
Dry fecal weight (g)	♀	1.00 ± 0.27	1.12 ± 0.11	0.84 ± 0.18	0.071	0.083	0.715	NS
	♂	0.88 ± 0.10	0.81 ± 0.12	0.98 ± 0.11				

Abbreviations: HET, heterozygous: Female *N* = 6, Male *N* = 6; HOM, homozygous: Female *N* = 6, Male *N* = 5; NS, Not significant; WT, Wild type: Female (♀) *N* = 6, Male (♂) *N* = 6.

**TABLE 2:** Metabolic cage balance results for *Fam111a* c.1450insA mice.

Day 3 (48–72 h) metabolic cage measurement	Sex	WT Mean ± SD	HET Mean ± SD	HOM Mean ± SD	Two‐way ANOVA interaction *p*‐value	Two‐way ANOVA sex *p*‐value	Two‐way ANOVA genotype *p*‐value	Šidák's multiple comparisons (Adjusted *p*‐value <0.05)
Body weight (g)	♀	27.90 ± 3.05	28.41 ± 2.04	30.20 ± 1.51	0.260	0.0001	0.727	♂ WT > ♀ WT ♂ HET > ♀ HET
	♂	33.44 ± 2.51	34.76 ± 2.39	32.54 ± 3.71				
Food intake (g)	♀	4.03 ± 0.39	4.17 ± 0.65	3.8 ± 0.57	0.545	0.096	0.229	NS
	♂	5.01 ± 1.19	4.61 **±** 0.78	3.96 **±** 0.93				
Water intake (mL)	♀	5.20 ± 0.80	4.96 ± 1.05	4.83 ± 0.42	0.544	0.998	0.999	NS
	♂	6.04 ± 0.98	4.86 ± 1.23	4.72 ± 1.46				
Urine volume (mL)	♀	1.42 ± 0.28	1.27 ± 0.17	1.46 ± 0.22	0.581	0.0006	0.599	♂ WT > ♀ WT ♂ HET > ♀ HET
	♂	2.51 ± 0.47	2.20 ± 0.53	2.01 ± 1.28				
Urine pH	♀	6.36 ± 0.48	6.20 ± 0.28	6.52 ± 0.46	0.566	0.924	0.950	NS
	♂	6.25 ± 0.10	6.50 ± 0.94	6.27 ± 0.12				
Dry fecal weight (g)	♀	0.90 ± 0.12	0.94 ± 0.20	0.95 ± 0.12	0.258	0.086	0.560	NS
	♂	1.22 ± 0.29	1.04 ± 0.22	0.95 ± 0.25				

Abbreviations: HET, heterozygous: Female *N* = 4, Male *N* = 6; HOM, homozygous: Female *N* = 5, Male *N* = 3; NS, Not significant; WT, Wild‐type: Female (♀) *N* = 5, Male (♂) *N* = 4.

### 3.1 Blood gas results and PTH

Blood gas results from HET and HOM *Fam111a* c.1253‐1464del mice were not different compared to WT mice (Table 3). However, male HET mice had greater blood chloride levels than female HET mice (Table 3). The hematocrit and hemoglobin levels were significantly higher in female HET mice compared to males (Table 3). Similarly, female HET and HOM *Fam111a* c.1450insA mice had significantly higher hematocrit and hemoglobin levels compared to males (Table 4). The carbon dioxide levels were significantly greater in female HOM c.1450insA mice compared to WT animals (Table 4).

**TABLE 3:** Blood gas results for *Fam111a* c.1253‐1464del mice.

Blood gas measurements	Sex	WT Mean ± SD	HET Mean ± SD	HOM Mean ± SD	Two‐way ANOVA interaction *p*‐value	Two‐way ANOVA sex *p*‐value	Two‐way ANOVA genotype *p*‐value	Šidák's multiple comparisons (Adjusted *p*‐value <0.05)
Ionized calcium (mM)	♀	1.04 ± 0.10	1.08 ± 0.04	1.11 ± 0.06	0. 174	0.348	0.310	NS
	♂	1.08 ± 0.05	1.01 ± 0.11	1.06 ± 0.01				
Sodium (mM)	♀	144.40 ± 1.67	145.67 ± 1.51	144.83 ± 0.98	0.403	0.578	0.851	NS
	♂	144.83 ± 1.94	144.33 ± 2.16	144.80 ± 0.84				
Potassium (mM)	♀	4.86 ± 0.84	4.75 ± 0.88	4.68 ± 0.31	0.880	0.246	0.955	NS
	♂	4.97 ± 0.20	5.12 ± 0.90	5.02 ± 0.48				
Chloride (mM)	♀	116.60 ± 0.55	115.83 ± 0.98	117.17 ± 1.33	0.073	0.003	0.763	♂ HET > ♀ HET
	♂	118.17 ± 2.48	119.67 ± 2.66	117.60 ± 0.89				
TCO_2_ (mM)	♀	22.80 ± 1.10	23.00 ± 1.41	23.17 ± 1.47	0.590	0.059	0.774	NS
	♂	22.50 ± 1.05	21.33 ± 2.88	21.80 ± 0.84				
Glucose (mg/dL)	♀	217.40 ± 20.16	209.67 ± 20.53	199.50 ± 30.85	0.489	0.505	0.343	NS
	♂	210.00 ± 17.37	224.67 ± 25.45	207.60 ± 15.34				
BUN (mg/dL)	♀	23.40 ± 1.14	24.33 ± 4.50	24.17 ± 1.72	0.942	0.234	0.844	NS
	♂	25.83 ± 1.47	27.67 ± 12.44	25.80 ± 4.09				
Hct (%PCV)	♀	37.60 ± 0.55	39.67 ± 2.66	36.67 ± 0.52	0.008	0.002	0.268	♀ HET > ♂ HET
	♂	37.17 ± 2.23	35.00 ± 1.67	36.00 ± 0.71				
Hb (g/dL)	♀	12.78 ± 0.16	13.50 ± 0.90	12.47 ± 0.21	0.007	0.002	0.242	♀ HET > ♂ HET
	♂	12.63 ± 0.74	11.90 ± 0.56	12.22 ± 0.25				

Abbreviations: BUN, blood urea nitrogen; Hb, hemoglobin; Hct, hematocrit; HET, heterozygous: female *N* = 6, male *N* = 6; HOM, homozygous: female *N* = 6, male *N* = 5; NS: not significant; TCO_2_, total carbon dioxide; WT, Wild type: female (♀) *N* = 6, male (♂) *N* = 6.

**TABLE 4:** Blood gas results for *Fam111a* c.1450insA mice.

Blood gas measurements	Sex	WT Mean ± SD	HET Mean ± SD	HOM Mean ± SD	Two‐way ANOVA Interaction *p*‐Value	Two‐way ANOVA Sex *p*‐Value	Two‐way ANOVA Genotype *p*‐Value	Šidák's multiple comparisons (Adjusted *p*‐Value <0.05)
Ionized calcium (mM)	♀	0.97 ± 0.11	1.03 ± 0.03	1.09 ± 0.06	0.138	0.773	0.982	NS
	♂	1.07 ± 0.06	1.06 ± 0.04	1.11 ± 0.05				
Sodium (mM)	♀	146.40 ± 1.67	145.75 ± 2.22	145.40 ± 2.07	0.612	0.684	0.201	NS
	♂	147.67 ± 1.16	145.17 ± 2.23	145.67 ± 0.58				
Potassium (mM)	♀	4.80 ± 0.26	4.85 ± 0.34	5.14 ± 0.94	0.568	0.197	0.875	NS
	♂	4.50 ± 0.53	4.80 ± 0.96	4.37 ± 0.32				
Chloride (mM)	♀	119.20 ± 2.59	117.50 ± 1.73	117.60 ± 1.34	0.858	0.937	0.120	NS
	♂	119.67 ± 2.52	117.83 ± 2.40	117.00 ± 0.00				
TCO_2_ (mM)	♀	19.60 ± 3.13	21.75 ± 0.50	24.40 ± 1.82	0.105	0.732	0.035	♀ WT < ♀ HOM
	♂	22.33 ± 4.73	19.40 ± 0.89	23.00 ± 1.73				
Glucose (mg/dL)	♀	182.40 ± 32.72	169.75 ± 23.42	208.80 ± 56.47	0.769	0.071	0.202	NS
	♂	207.00 ± 23.64	215.33 ± 13.11	234.00 ± 37.64				
BUN (mg/dL)	♀	27.00 ± 2.00	25.25 ± 6.75	21.60 ± 3.21	0.844	0.626	0.137	NS
	♂	29.33 ± 3.22	28.00 ± 11.73	20.67 ± 4.73				
Hct (%PCV)	♀	38.00 ± 2.00	39.25 ± 2.63	40.40 ± 1.14	0.926	0.0003	0.149	♀ HET > ♂ HET ♀ HOM > ♂ HOM
	♂	35.00 ± 3.00	35.67 ± 1.63	36.67 ± 0.58				
Hb (g/dL)	♀	12.92 ± 0.68	13.35 ± 0.88	13.74 ± 0.38	0.924	0.0003	0.145	♀ HET > ♂ HET ♀ HOM > ♂ HOM
	♂	11.90 ± 1.00	12.13 ± 0.57	12.47 ± 0.23				

Abbreviations: BUN, blood urea nitrogen; Hb, hemoglobin; Hct, hematocrit; HET, heterozygous: female *N* = 4, Male *N* = 6; HOM, homozygous: Female *N* = 5, Male *N* = 3; NS, Not significant; TCO_2_, total carbon dioxide; WT, Wild type: female (♀) *N* = 5, male (♂) *N* = 4.

**FIGURE 2:**
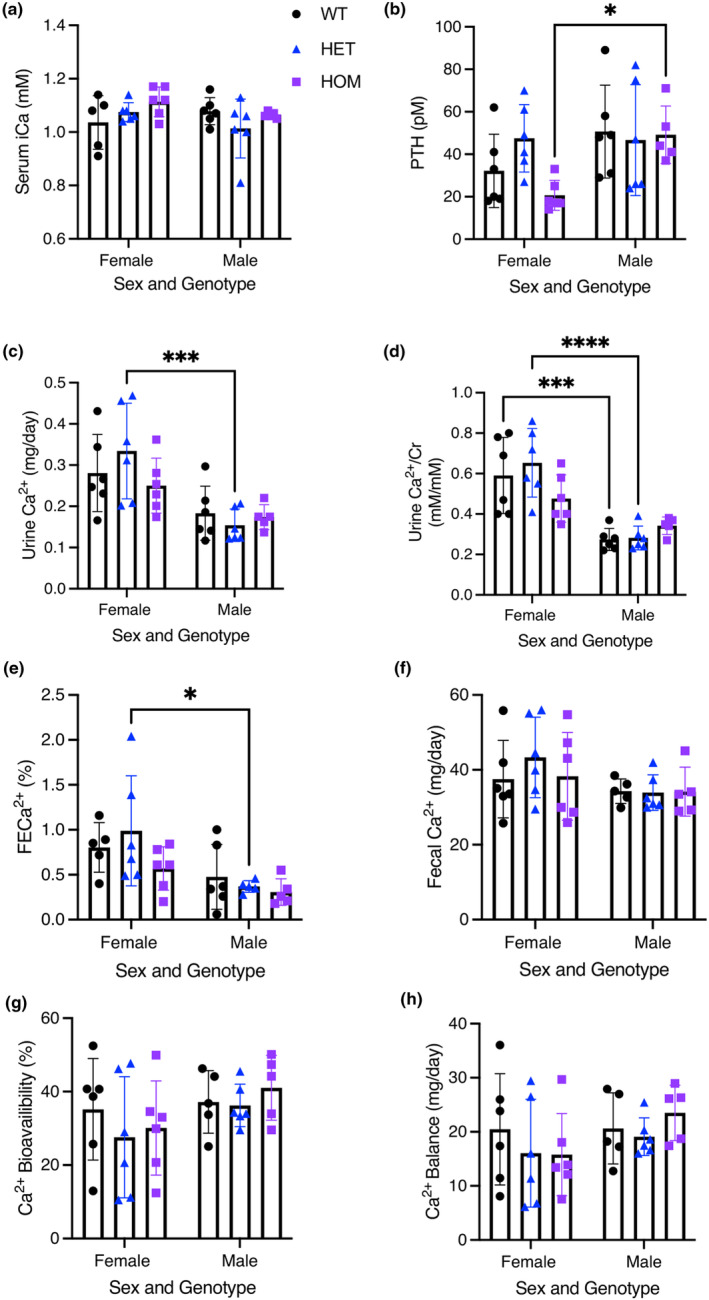
Calcium handling of WT and c.1253‐1464del (HET and HOM) mice for (a) serum ionized Ca^2+^ (iCa), (b) serum parathyroid hormone (PTH), (c) 24 hr urine Ca^2+^ excretion, (d) urine Ca^2+^ to creatinine (Cr) ratio, (E) fractional excretion of Ca^2+^ (FECa^2+^), (f) fecal Ca^2+^, (g) Ca^2+^ bioavailability, and (h) Ca^2+^ balance. Data are presented as mean ± SD. Female sample size: *N* = 5 WT, *N* = 6 HET, *N* = 6 HOM. Male sample size: *N* = 6 WT, *N* = 6 HET, *N* = 5 HOM. A two‐way ANOVA was used to determine the effects of sex and genotype on a variable. Multiple comparisons were made with Šidák's test and significance is denoted by an asterisk (* *p* < 0.05, *** *p* < 0.001, **** *p* < 0.0001).

**FIGURE 3:**
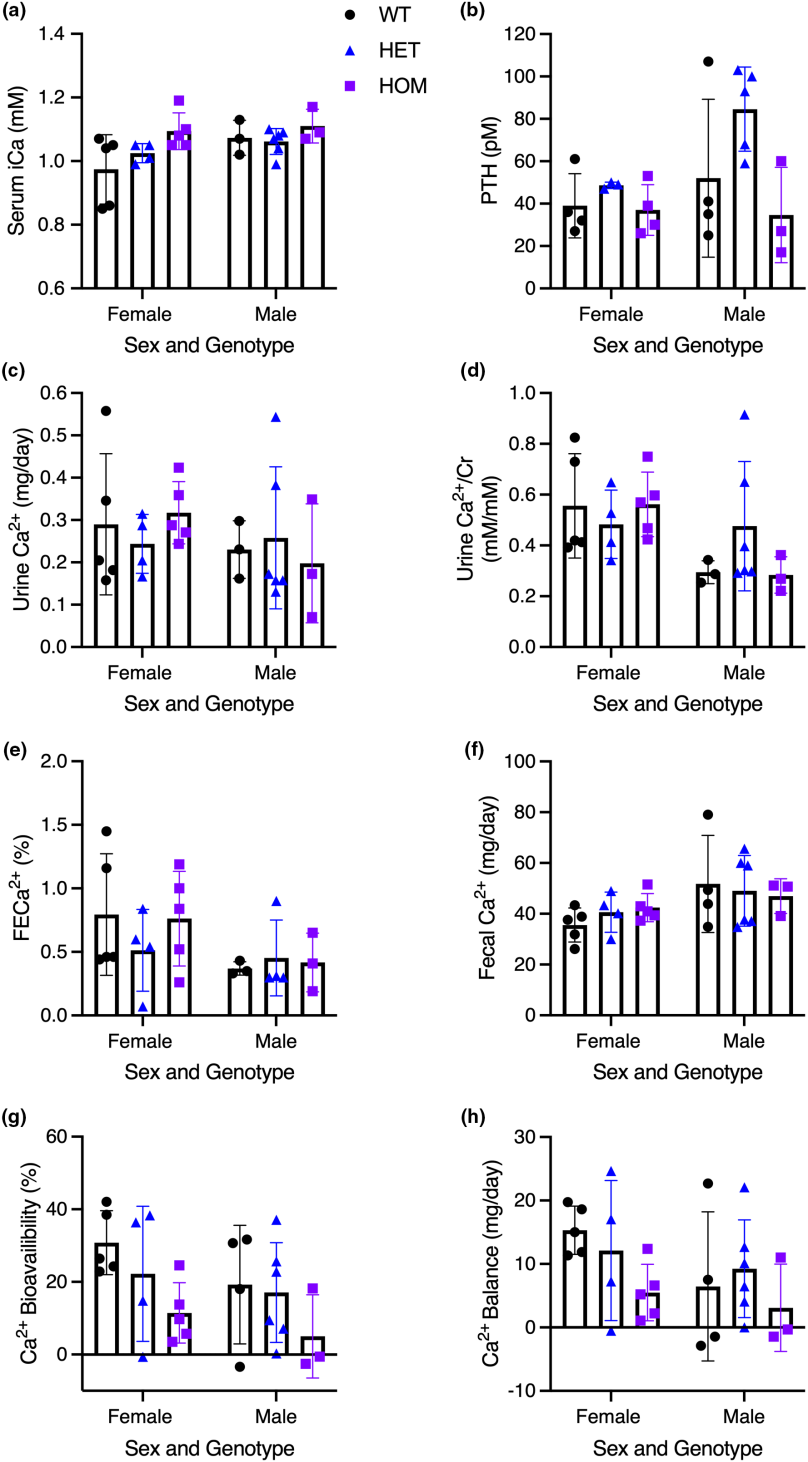
Calcium handling of WT and c.1450insA (HET and HOM) mice for (a) Serum ionized Ca^2+^ (iCa), (b) Serum parathyroid hormone (PTH), (c) 24 h urine Ca^2+^ excretion, (d) Urine Ca^2+^ to creatinine (Cr) ratio, (e) Fractional excretion of Ca^2+^ (FECa^2+^), (f) Fecal Ca^2+^, (g) Ca^2+^ bioavailability, and (h) Ca^2+^ balance. Data are presented as mean ± SD. Female sample size: *N* = 5 WT, *N* = 4 HET, *N* = 5 HOM. Male sample size: *N* = 3 WT, *N* = 6 HET, *N* = 3 HOM. A two‐way ANOVA was used to determine the effects of sex and genotype on a variable. Multiple comparisons were made with Šidák's test.

A central feature of KCS and OCS is hypocalcemia and hypoparathyroidism. However, blood ionized Ca^2+^ levels were not different between HET or HOM mice carrying either mutation and WT mice of the same sex. Similarly, there were no significant differences in serum PTH levels between HET or HOM mice with either mutation compared to WT mice of the same sex (Figures 2b and 3b). PTH was, however, significantly higher in male HOM *Fam111a* c.1253‐1464del mice compared to female HOM animals (Figure 2b).

### 3.2 Urinary and fecal calcium excretion

We next examined urinary Ca^2+^ excretion and measured total 24‐hour Ca^2+^ excretion, Ca^2+^ excretion normalized to creatinine, and fractional excretion of Ca^2+^ (FECa^2+^). We failed to detect a significant difference in urinary Ca^2+^ excretion between HET or HOM mice with either mutation and WT mice of the same sex by any measurement of Ca^2+^ excretion (Figures 2c–e and 3c–e). However, female HET *Fam111a* c.1253‐1464del mice had greater urinary Ca^2+^ excretion compared to male HET mice (Figure 2c–e). Female WT mice also had higher urine Ca^2+^/Cr than male WT mice (Figure 2d). We also measured the excretion of other common cations and anions found in urine, as well as creatinine (Tables 5 and 6). There were no significant differences between HET or HOM mice with either mutation compared to WT mice (Tables 5 and 6). The only sex differences observed were that female WT and HET *Fam111a* c.1253‐1464del mice had higher urine K^+^/Cr compared to males (Table 5). Additionally, female WT mice had higher urine Na^+^/Cr and FECl^−^ compared to males (Table 5). Urine creatinine was higher in male WT mice than in female WT mice (Table 6).

**TABLE 5:** Urine results for *Fam111a* c.1253‐1464del mice.

Urine ion and Cr excretion	Sex	WT Mean ± SD	*N*	HET Mean ± SD	*N*	HOM Mean ± SD	*N*	Two‐way ANOVA interaction *p*‐value	Two‐way ANOVA sex *p*‐value	Two‐way ANOVA genotype *p*‐value	Šidák's Multiple comparisons (Adjusted *p*‐value <0.05)
Ca^2+^ (mg/day)	♀	0.28 ± 0.09	6	0.33 ± 0.12	6	0.25 ± 0.07	6	0.233	<0.0001	0.599	♀ HET > ♂ HET
	♂	0.18 ± 0.07	6	0.15 ± 0.04	6	0.17 ± 0.03	5				
Mg^2+^ (mg/day)	♀	1.39 ± 0.42	6	1.26 ± 0.63	6	1.07 ± 0.17	6	0.104	0.947	0.349	NS
	♂	1.30 ± 0.51	6	0.96 ± 0.21	6	1.49 ± 0.15	5				
K^+^ (mg/day)	♀	40.51 ± 11.05	6	42.25 ± 7.92	6	36.16 ± 7.50	6	0.151	0.177	0.836	NS
	♂	37.30 ± 9.25	6	31.93 ± 4.40	6	38.82 ± 1.29	5				
Na^+^ (mg/day)	♀	10.12 ± 2.23	6	9.63 ± 2.46	6	9.52 ± 2.60	6	0.676	0.529	0.429	NS
	♂	10.06 ± 2.57	6	8.28 ± 0.89	6	9.56 ± 0.86	5				
Cl^−^(mg/day)	♀	24.18 ± 9.71	6	27.20 ± 13.75	6	28.67 ± 6.71	6	0.594	0.102	0.452	NS
	♂	18.68 ± 10.13	6	25.80 ± 3.10	6	18.89 ± 11.58	5				
PO_4_^3−^(mg/day)	♀	8.69 ± 4.53	6	6.31 ± 3.69	6	10.98 ± 0.24	5	0.041	0.471	0.943	NS
	♂	10.37 ± 5.97	6	11.87 ± 2.58	6	6.91 ± 5.27	5				
Ca^2+^/Cr (mM/mM)	♀	0.59 ± 0.19	6	0.65 ± 0.17	6	0.48 ± 0.12	6	0.069	<0.0001	0.519	♀ WT > ♂ WT ♀ HET > ♂ HET
	♂	0.27 ± 0.05	6	0.28 ± 0.06	6	0.34 ± 0.04	5				
Mg^2+^/Cr (mM/mM)	♀	4.82 ± 1.40	6	4.22 ± 2.17	6	3.37 ± 0.52	6	0.009	0.318	0.517	NS
	♂	3.11 ± 0.49	6	2.93 ± 0.79	6	5.02 ± 1.60	5				
K^+^/Cr (mM/mM)	♀	86.46 ± 20.01	6	85.82 ± 6.47	6	70.30 ± 9.91	6	0.001	0.001	0.823	♀ WT > ♂ WT ♀ HET > ♂ HET
	♂	57.46 ± 6.65	6	60.30 ± 9.94	6	80.01 ± 15.90	5				
Na^+^/Cr (mM/mM)	♀	36.71 ± 5.17	6	32.98 ± 3.15	6	31.31 ± 6.37	6	0.012	0.004	0.434	♀ WT > ♂ WT
	♂	26.35 ± 3.26	6	26.63 ± 3.86	6	33.24 ± 5.33	5				
Cl^−^/Cr(mM/mM)	♀	56.97 ± 18.71	6	60.50 ± 27.00	6	61.52 ± 11.50	6	0.493	0.055	0.312	NS
	♂	34.08 ± 19.66	6	54.03 ± 10.43	6	39.83 ± 18.73	5				
PO_4_^3−^/Cr (mM/mM)	♀	7.93 ± 4.53	6	5.14 ± 2.52	6	8.58 ± 0.76	5	0.041	0.972	0.868	NS
	♂	7.35 ± 4.48	6	9.08 ± 0.87	6	5.34 ± 3.37	5				
FECa^2+^ (%)	♀	0.80 ± 0.28	5	0.99 ± 0.61	6	0.57 ± 0.24	6	0.451	0.003	0.238	♀ HET > ♂ HET
	♂	0.48 ± 0.36	6	0.37 ± 0.07	5	0.31 ± 0.15	5				
FENa^+^ (%)	♀	0.38 ± 0.16	5	0.29 ± 0.05	5	0.27 ± 0.08	6	0.837	0.432	0.204	NS
	♂	0.32 ± 0.20	6	0.29 ± 0.10	6	0.22 ± 0.11	5				
FEK^+^ (%)	♀	25.32 ± 8.66	5	27.21 ± 7.25	6	19.38 ± 6.01	6	0.747	0.090	0.311	NS
	♂	20.88 ± 14.84	6	18.45 ± 3.99	6	16.13 ± 9.79	5				
FECl^−^ (%)	♀	0.85 ± 0.49	5	0.71 ± 0.22	6	0.67 ± 0.21	6	0.107	0.006	0.138	♀ WT > ♂ WT
	♂	0.40 ± 0.22	6	0.71 ± 0.25	6	0.29 ± 0.10	5				
Cr (μmol/day)	♀	12.45 ± 1.97	6	12.18 ± 2.44	6	13.10 ± 1.45	6	0.714	0.200	0.967	NS
	♂	13.93 ± 3.62	6	13.73 ± 2.25	6	13.20 ± 1.56	5				

Abbreviations: ♀, female; ♂, male; Cr, creatinine; HET, heterozygous; HOM, homozygous; NS, not significant; WT, Wild type.

**TABLE 6:** Urine results for *Fam111a* c.1450insA mice.

Urine ion and Cr excretion	Sex	WT Mean ± SD	*N*	HET Mean ± SD	*N*	HOM Mean ± SD	*N*	Two‐way ANOVA interaction *p*‐value	Two‐way ANOVA sex *p*‐value	Two‐way ANOVA genotype *p*‐value	Šidák's multiple comparisons (Adjusted *p*‐value <0.05)
Ca^2+^ (mg/day)	♀	0.29 ± 0.17	5	0.24 ± 0.07	4	0.32 ± 0.07	5	0.576	0.309	0.988	NS
	♂	0.23 ± 0.07	3	0.26 ± 0.17	6	0.20 ± 0.14	3				
Mg^2+^ (mg/day)	♀	1.03 ± 0.52	5	1.25 ± 0.32	4	0.91 ± 0.80	5	0.248	0.131	0.705	NS
	♂	1.81 ± 0.61	4	1.03 ± 0.80	6	1.64 ± 1.03	3				
K^+^ (mg/day)	♀	36.10 ± 5.18	5	33.85 ± 7.55	4	39.37 ± 7.23	5	0.206	0.039	0.160	NS
	♂	57.34 ± 1.89	3	38.61 ± 6.75	6	42.22 ± 28.10	3				
Na^+^ (mg/day)	♀	8.06 ± 1.10	5	8.55 ± 2.40	4	9.60 ± 1.95	5	0.232	0.157	0.465	NS
	♂	12.27 ± 3.92	4	8.71 ± 1.36	6	9.94 ± 5.59	3				
Cl^−^ (mg/day)	♀	27.38 ± 1.83	4	29.06 ± 5.13	4	31.91 ± 5.62	5	0.232	0.058	0.439	NS
	♂	44.22 ± 1.65	3	31.14 ± 5.47	6	35.17 ± 23.76	3				
PO_4_^3−^ (mg/day)	♀	6.76 ± 3.96	5	6.37 ± 2.98	4	8.26 ± 4.60	5	0.703	0.142	0.269	NS
	♂	10.14 ± 4.57	4	6.92 ± 0.60	5	11.30 ± 5.09	3				
Ca^2+^/Cr (mM/mM)	♀	0.56 ± 0.21	5	0.48 ± 0.13	4	0.56 ± 0.13	5	0.225	0.053	0.753	NS
	♂	0.29 ± 0.04	3	0.48 ± 0.25	6	0.28 ± 0.07	3				
Mg^2+^/Cr (mM/mM)	♀	3.75 ± 2.40	5	4.11 ± 1.09	4	2.61 ± 2.28	5	0.419	0.998	0.940	NS
	♂	3.27 ± 0.12	3	3.17 ± 2.35	6	4.03 ± 0.57	3				
K^+^/Cr (mM/mM)	♀	75.85 ± 10.57	5	69.10 ± 16.57	4	70.87 ± 7.43	5	0.221	0.430	0.578	NS
	♂	66.13 ± 10.17	4	76.09 ± 8.40	6	63.31 ± 12.20	3				
Na^+^/Cr (mM/mM)	♀	28.76 ± 3.55	5	29.71 ± 8.86	4	29.43 ± 4.11	5	0.869	0.382	0.743	NS
	♂	26.94 ± 6.71	4	29.24 ± 3.14	6	26.33 ± 1.89	3				
Cl^−^/Cr (mM/mM)	♀	68.79 ± 7.45	5	65.37 ± 12.34	4	63.48 ± 7.32	5	0.273	0.213	0.459	NS
	♂	57.31 ± 6.95	4	67.79 ± 8.78	6	58.89 ± 11.61	3				
PO_4_^3−^/Cr (mM/mM)	♀	6.20 ± 3.61	5	5.34 ± 2.58	4	6.12 ± 3.37	5	0.676	0.922	0.434	NS
	♂	5.44 ± 2.20	4	4.98 ± 1.35	6	7.54 ± 0.51	3				
FECa^2+^ (%)	♀	0.79 ± 0.48	5	0.51 ± 0.32	4	0.76 ± 0.37	5	0.561	0.072	0.795	NS
	♂	0.37 ± 0.05	3	0.45 ± 0.30	4	0.42 ± 0.23	3				
FENa^+^ (%)	♀	0.23 ± 0.02	4	0.23 ± 0.14	4	0.31 ± 0.15	5	0.711	0.840	0.505	NS
	♂	0.22 ± 0.03	3	0.29 ± 0.13	5	0.28 ± 0.11	3				
FEK^+^ (%)	♀	20.29 ± 4.20	5	15.05 ± 8.97	4	21.51 ± 10.43	5	0.539	0.450	0.688	NS
	♂	19.18 ± 3.86	3	22.46 ± 7.36	5	22.74 ± 10.07	3				
FECl^−^ (%)	♀	0.74 ± 0.14	5	0.58 ± 0.33	4	0.82 ± 0.37	5	0.452	0.765	0.716	NS
	♂	0.62 ± 0.04	3	0.84 ± 0.40	5	0.79 ± 0.38	3				
Cr (μmol/day)	♀	12.00 ± 3.08	5	12.00 ± 1.63	4	14.00 ± 1.41	5	0.244	0.026	0.207	♂ WT > ♀ WT
	♂	18.25 ± 3.30	4	13.17 ± 1.83	6	16.00 ± 3.10	3				

Abbreviations: ♀, female; ♂, male; Cr, creatinine; HET, heterozygous; HOM, homozygous; NS, not significant; WT, Wild type.

To assess intestinal absorption of Ca^2+^, we collected feces and measured Ca^2+^ content (Figures 2f,h and 3f,h). This enabled us to calculate total fecal Ca^2+^ excretion, intestinal Ca^2+^ absorption, and overall Ca^2+^ balance. Again, we failed to detect a significant difference between either HET or HOM mice harboring either mutation and WT mice of the same sex and genotype. Together these data strongly suggest that the serine protease domain of *Fam111a* is dispensable for Ca^2+^ homeostasis.

### 3.3 Micro‐CT scans of bone

**FIGURE 4:**
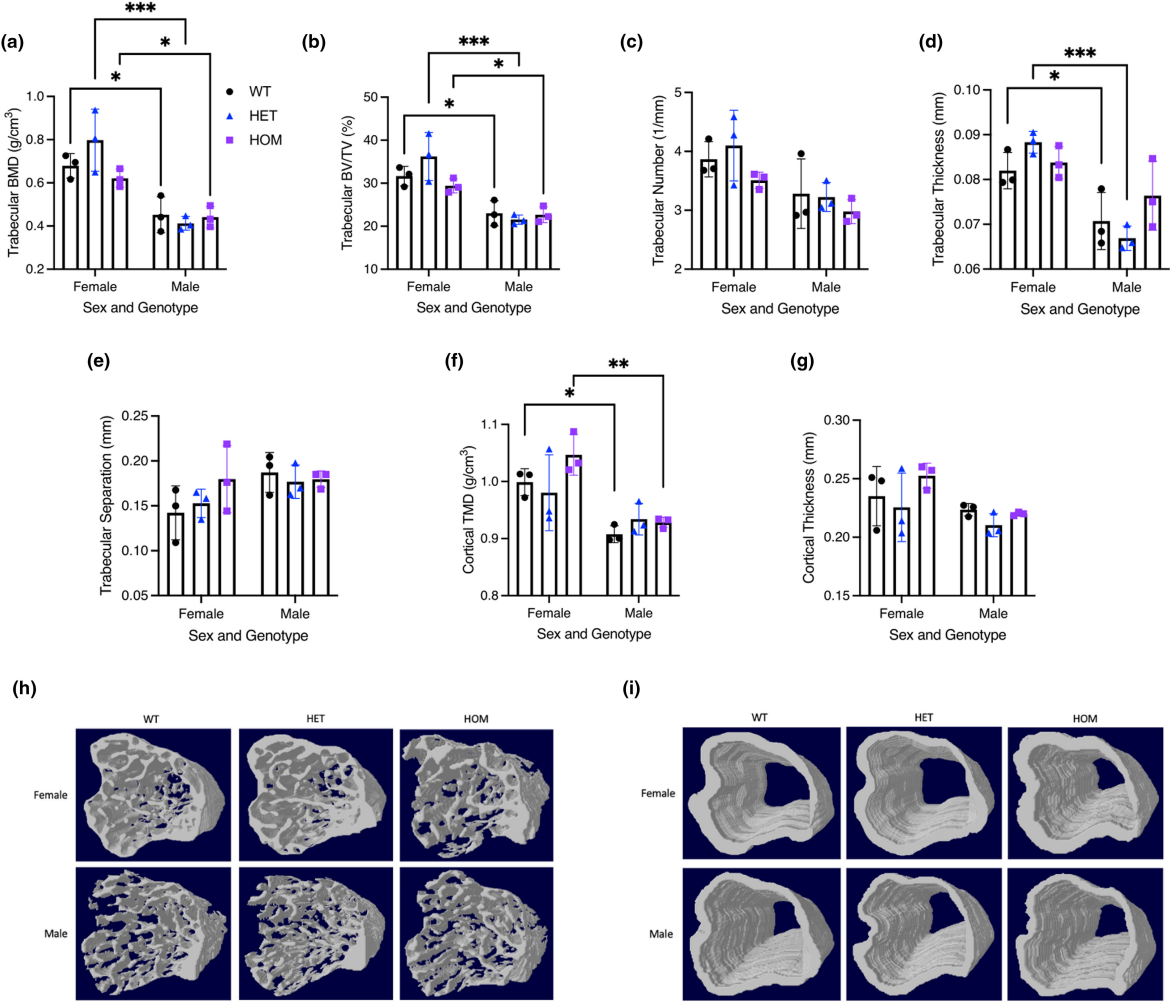
Micro‐CT analysis of tibia from 16‐week‐old female and male WT and c.1253‐1464del (HET and HOM) mice. (a) Trabecular bone mineral density (BMD), (b) trabecular bone volume/tissue volume (BV/TV), (c) trabecular number, (d) trabecular thickness, (e) trabecular separation, (f) cortical tissue mineral density (TMD), (g) cortical thickness, (h) 3D images of the trabecular bone, (i) 3D images of the cortical bone. Data are presented as mean ± SD. Sample size is *N* = 3 for each genotype. A two‐way ANOVA was used to determine the effects of sex and genotype on a variable. Multiple comparisons were made with Šidák's test and significance is denoted by an asterisk (* *p* < 0.05, ** *p* < 0.01, *** *p* < 0.001).

**FIGURE 5:**
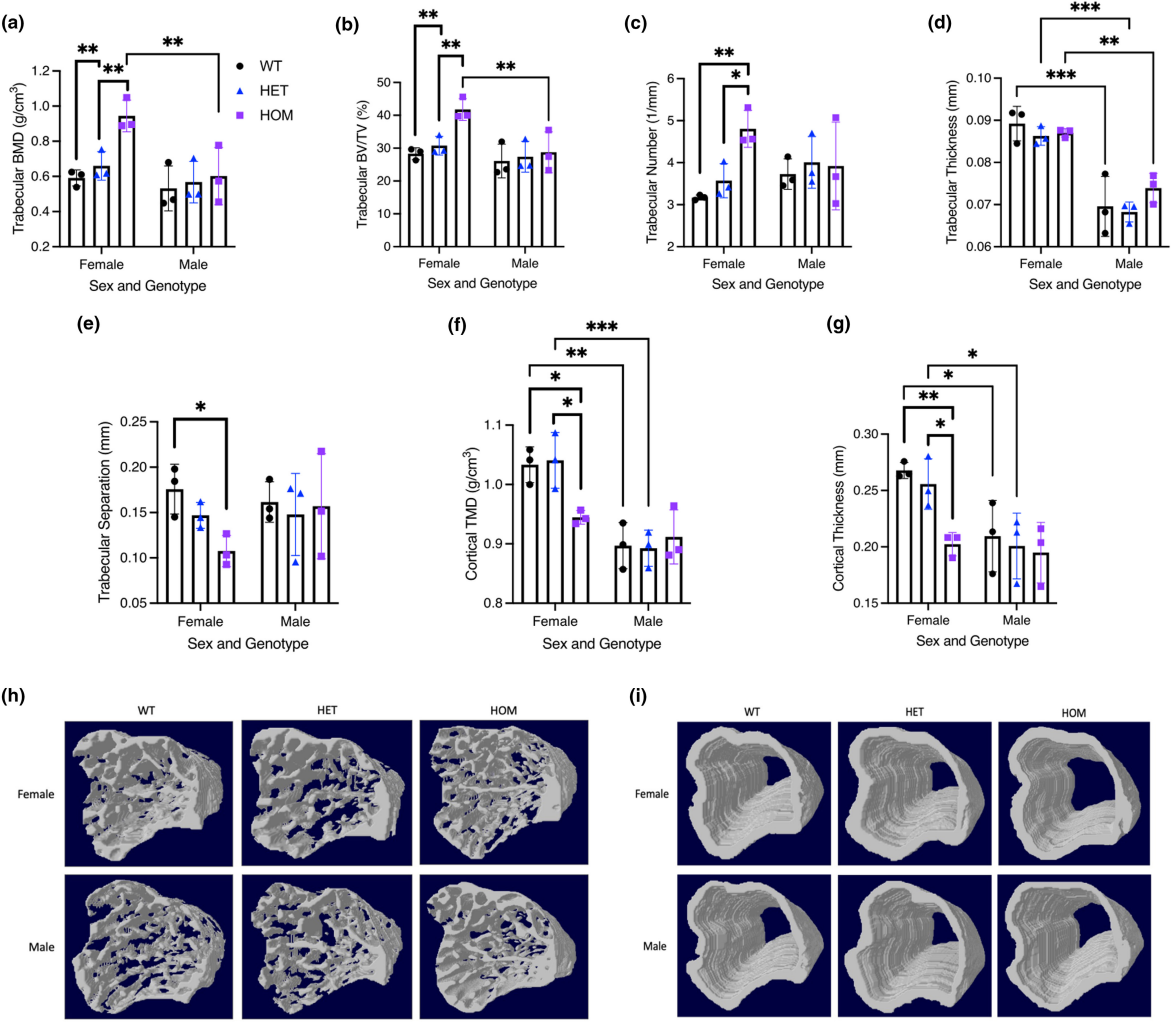
Micro‐CT analysis of tibia from 16‐week‐old female and male WT and c.1450insA (HET and HOM) mice. (a) Trabecular bone mineral density (BMD), (b) trabecular bone volume/tissue volume (BV/TV), (c) trabecular number, (d) trabecular thickness, (e) trabecular separation, (f) cortical tissue mineral density (TMD), (g) cortical thickness, (h) 3D images of the trabecular bone, (i) 3D images of the cortical bone. Data are presented as mean ± SD. Sample size is *N* = 3 for each genotype. A two‐way ANOVA was used to determine the effects of sex and genotype on a variable. Multiple comparisons were made with Šidák's test and significance is denoted by an asterisk (* *p* < 0.05, ** *p* < 0.01, *** *p* < 0.001).

There was no significant difference in bone morphology or mineral density between HET or HOM *Fam111a* c.1253‐1464del mice compared to WT mice of the same sex (Figure 4). However, compared to females, male WT, HET, and HOM had significantly lower trabecular bone mineral density (BMD) and percent bone volume/tissue volume (BV/TV). WT and HET males had lower trabecular thickness compared to females and cortical tissue mineral density (TMD) was lower in male WT and HOM animals. Similarly, there were no significant differences in bone mineral parameters or density between male *Fam111a* c.1450insA HET or HOM mice compared to WT mice (Figure 5). In contrast, female *Fam111a* c.1450insA HOM mice had significantly greater trabecular BMD, percent BV/TV, and trabecular number compared to HET animals. Further, female *Fam111a* c.1450insA HOM mice had significantly lower trabecular separation than WT mice and lower cortical TMD and cortical thickness compared to both WT and or HET mice. Compared to females, male WT, HET, and HOM *Fam111a* c.1450insA mice had lower trabecular thickness. Also, male HOM mice had lower trabecular BMD and percent BV/TV, while both male WT and HET had lower cortical TMD and cortical thickness.

## 4 DISCUSSION

All mutations identified thus far that cause KCS and OCS are located within the c‐terminal serine protease domain. We therefore hypothesized that disruption of this domain would result in a similar phenotype in mice, including hypocalcemia, hypoparathyroidism, and altered bone morphology. However, we found that a frameshift insertion (c.1450insA) or large deletion (c.1253‐1464del) in the c‐terminal, serine protease domain of *Fam111a* in mice did not lead to a phenotype resembling that of KCS or OCS patients. The mutant mice did not have hypocalcemia nor lower serum PTH. Additionally, Ca^2+^ homeostasis as assessed by urine and fecal Ca^2+^ excretion, intestinal Ca^2+^ absorption, and overall Ca^2+^ balance, was not different between mutant and WT mice. Another characteristic of KCS and OCS is thin, short, but dense bones (Rosato et al., 2022; Unger et al., 2013), however, the tibia of HET mice with either the c.1450insA or c.1253‐1464del frameshift mutation had normal mineral density and microarchitecture. The tibia of female HOM mice with the c.1450insA mutation did display greater trabecular BMD and lower cortical TMD compared to WT. Perhaps the insertion in Fam111a results in a misfolded protein leading to these subtle alterations in female mice, due to their greater propensity for bone disease relative to males. On the other hand, HET animals were not different from WT mice and in humans, a single affected allele is sufficient to cause disease. Moreover, both males and females appear to be equally affected by KCS and OCS. Thus, disruption of the c‐terminal serine protease domain in mice does not phenocopy the human disease.

The mice in our study had mutations, which resulted in premature stop codons. They are thus nonsense mutations leading to a truncated protein that is non‐functional. Ilenwabor and colleagues (Ilenwabor et al., 2022) also found that mice with knockout (KO) of the protein‐coding region of *Fam111a* (Exons 3 and 4) also had normal serum PTH levels, no difference in electrolyte homeostasis, and normal bone morphology. They concluded that *Fam111a* was dispensable for electrolyte homeostasis in mice. Consistent with their results we also found no differences in Ca^2+^ homeostasis in both mouse models with neither displaying characteristics of KSC or OCS.

Why the deletion of *Fam111a* (Ilenwabor et al., 2022) nor the disruption of the serine protease domain does not result in a KCS or OCS phenotype is unclear. Perhaps in mice, but not humans, there is another protease that compensates for the loss of *Fam111a*. Consistent with this, there are species differences between humans and mice in relation to *Fam111a*. In humans, *FAM111A* is located on chromosome 11 whereas it is located on chromosome 19 in mice. Alignment of the protein sequences shows that the proteins are only 56.59% conserved. An additional species difference includes the presence of a gene called *FAM111B* in humans, but not in mice. It is homologous to *FAM111A* and also has a trypsin‐like cysteine/serine protease domain in the C‐terminus. *FAM111B* is located only 16 kb upstream of *FAM111A* on human chromosome 11. Although the FAM111A and FAM111B proteins are known to form a complex, FAM111A was not found to be dependent on FAM111B for its effect on DNA‐associated processes (Hoffmann et al., 2020). However, it has yet to be tested whether the association between FAM111A and FAM111B plays a role in Ca^2+^ homeostasis in humans. Unfortunately, as in this case, mouse models do not always phenocopy human disease (Barbaric et al., 2007; Perlman, 2016).

Importantly, humans with KCS or OCS do not harbor mutations that cause a frameshift, large deletions, or early terminations (Rosato et al., 2022; Unger et al., 2013). Instead, they have single amino acid changes, i.e. substitutions or deletions. Our mouse model may not have generated a KCS or OCS phenotype because the mutations introduced caused large structural changes through a frameshift or early stop codon, disrupting protein function or a protein–protein interaction. A gain‐of‐function mechanism has been proposed for the human disease‐causing mutations which increase FAM111A protease activity, undermining cellular fitness by compromising nuclear barrier function and disrupting DNA replication (Hoffmann et al., 2020; Kojima et al., 2020; Nie et al., 2021). However, hyperactivation of FAM111A could cause an overall loss of function by increasing the autocleavage activity of FAM111A (Kojima et al., 2020). It is unclear whether the gain of function or loss of function of FAM111A causes the phenotype of KCS or OCS. Nevertheless, deletion of the entire protein‐coding region of the *Fam111a* gene (Ilenwabor et al., 2022) or disruption of the protease domain as described herein does not result in a KCS or OCS phenotype in mice.

## 5 SUMMARY/CONCLUSIONS

KCS and OCS are characterized by dysregulation in Ca^2+^ homeostasis, specifically hypocalcemia, low serum PTH, and skeletal abnormalities. Given that all mutations causing KCS or OCS are in the protease domain of FAM111A, we predicted that any disruption of this domain would negatively impact Ca^2+^ homeostasis. However, mice with a frameshift insertion or large deletion that disrupts the serine protease domain of *Fam111a* did not result in a KCS or OCS phenotype in mice. This could be due to compensation by another protease, secondary to a species difference between humans and mice, or because the specific mutations we introduced caused a loss of function instead of a gain of function of FAM111A. Future studies should introduce known KCS and OCS mutations into an animal model or use human cell models to delineate the molecular mechanism of how *FAM111A* mutations affect Ca^2+^ homeostasis.

## AUTHOR CONTRIBUTIONS

RSGT wrote the first draft of the manuscript. RTA, RSGT, CHLL, WP, SW, and MRD reviewed and edited the manuscript. RTA and RSGT designed experiments. RSGT, CHLL, SW, and WP performed experiments.

## FUNDING INFORMATION

Mouse transgenesis was performed at the University of Alberta Faculty of Medicine & Dentistry Transgenic Core, RRID:SCR_019175, which receives financial support from the Faculty of Medicine & Dentistry, University of Alberta, and Canada Foundation for Innovation (CFI) awards to contributing investigators. RSGT is funded by the Alberta Innovates Graduate Student Scholarship and Women and Children's Health Research Institute (WCHRI) Graduate Studentship Award. RTA is the Canada Research Chair in Renal Tubular Epithelial Transport Physiology. This work was funded by a grant from the Canadian Institutes of Health Research (CIHR, PS 166072).

## CONFLICT OF INTEREST STATEMENT

The author(s) declare no potential conflicts of interest with respect to the research, authorship, and/or publication of this article.

## Data Availability

All data is included in this manuscript.

## References

[phy215977-bib-0001] Alabert, C. , Bukowski‐Wills, J. C. , Lee, S. B. , Kustatscher, G. , Nakamura, K. , de Lima, A. F. , Menard, P. , Mejlvang, J. , Rappsilber, J. , & Groth, A. (2014). Nascent chromatin capture proteomics determines chromatin dynamics during DNA replication and identifies unknown fork components. Nature Cell Biology, 16, 281–293. 24561620 10.1038/ncb2918 PMC4283098

[phy215977-bib-0002] Barbaric, I. , Miller, G. , & Dear, T. N. (2007). Appearances can be deceiving: Phenotypes of knockout mice. Briefings in Functional Genomics & Proteomics, 6, 91–103. 17584761 10.1093/bfgp/elm008

[phy215977-bib-0003] Beggs, M. R. , Young, K. , Pan, W. , O'Neill, D. D. , Saurette, M. , Plain, A. , Rievaj, J. , Doschak, M. R. , Cordat, E. , Dimke, H. , & Alexander, R. T. (2021). Claudin‐2 and claudin‐12 form independent, complementary pores required to maintain calcium homeostasis. Proceedings of the National Academy of Sciences of the United States of America, 118, e2111247118. 34810264 10.1073/pnas.2111247118 PMC8694054

[phy215977-bib-0004] Dimke, H. , Desai, P. , Borovac, J. , Lau, A. , Pan, W. , & Alexander, R. T. (2013). Activation of the Ca(2+)‐sensing receptor increases renal claudin‐14 expression and urinary Ca(2+) excretion. American Journal of Physiology. Renal Physiology, 304, F761–F769. 23283989 10.1152/ajprenal.00263.2012 PMC4959880

[phy215977-bib-0005] Ferreira, P. G. , van Megen, W. H. , Tan, R. , Lee, C. H. L. , Svenningsen, P. , Alexander, R. T. , & Dimke, H. (2021). Renal claudin‐14 expression is not required for regulating Mg. American Journal of Physiology. Renal Physiology, 320, F897–F907. 33818126 10.1152/ajprenal.00590.2020

[phy215977-bib-0006] Fine, D. A. , Rozenblatt‐Rosen, O. , Padi, M. , Korkhin, A. , James, R. L. , Adelmant, G. , Yoon, R. , Guo, L. , Berrios, C. , Zhang, Y. , Calderwood, M. A. , Velmurgan, S. , Cheng, J. , Marto, J. A. , Hill, D. E. , Cusick, M. E. , Vidal, M. , Florens, L. , Washburn, M. P. , … DeCaprio, J. A. (2012). Identification of FAM111A as an SV40 host range restriction and adenovirus helper factor. PLoS Pathogens, 8, e1002949. 23093934 10.1371/journal.ppat.1002949 PMC3475652

[phy215977-bib-0007] Hoffmann, S. , Pentakota, S. , Mund, A. , Haahr, P. , Coscia, F. , Gallo, M. , Mann, M. , Taylor, N. M. , & Mailand, N. (2020). FAM111 protease activity undermines cellular fitness and is amplified by gain‐of‐function mutations in human disease. EMBO Reports, 21, e50662. 32776417 10.15252/embr.202050662 PMC7534640

[phy215977-bib-0008] Ilenwabor, B. P. , Schigt, H. , Kompatscher, A. , Bos, C. , Zuidscherwoude, M. , van der Eerden, B. C. J. , Hoenderop, J. G. J. , & de Baaij, J. H. F. (2022). FAM111A is dispensable for electrolyte homeostasis in mice. Scientific Reports, 12, 10211. 35715480 10.1038/s41598-022-14054-8 PMC9205974

[phy215977-bib-0009] Jiang, F. , & Doudna, J. A. (2017). CRISPR‐Cas9 structures and mechanisms. Annual Review of Biophysics, 46, 505–529. 10.1146/annurev-biophys-062215-010822 28375731

[phy215977-bib-0010] Kang, M. S. , Ryu, E. , Lee, S. W. , Park, J. , Ha, N. Y. , Ra, J. S. , Kim, Y. J. , Kim, J. , Abdel‐Rahman, M. , Park, S. H. , Lee, K. Y. , Kim, H. , Kang, S. , & Myung, K. (2019). Regulation of PCNA cycling on replicating DNA by RFC and RFC‐like complexes. Nature Communications, 10, 2420. 10.1038/s41467-019-10376-w PMC6546911 31160570

[phy215977-bib-0011] Kojima, Y. , Machida, Y. , Palani, S. , Caulfield, T. R. , Radisky, E. S. , Kaufmann, S. H. , & Machida, Y. J. (2020). FAM111A protects replication forks from protein obstacles via its trypsin‐like domain. Nature Communications, 11, 1318. 10.1038/s41467-020-15170-7 PMC7067828 32165630

[phy215977-bib-0012] Nie, M. , Oravcová, M. , Jami‐Alahmadi, Y. , Wohlschlegel, J. A. , Lazzerini‐Denchi, E. , & Boddy, M. N. (2021). FAM111A induces nuclear dysfunction in disease and viral restriction. EMBO Reports, 22, e50803. 33369867 10.15252/embr.202050803 PMC7857424

[phy215977-bib-0013] Perlman, R. L. (2016). Mouse models of human disease: An evolutionary perspective. Evolution, Medicine, and Public Health, 2016, 170–176. 27121451 10.1093/emph/eow014 PMC4875775

[phy215977-bib-0014] Plain, A. , Pan, W. , O'Neill, D. , Ure, M. , Beggs, M. R. , Farhan, M. , Dimke, H. , Cordat, E. , & Alexander, R. T. (2020). Claudin‐12 knockout mice demonstrate reduced proximal tubule calcium permeability. International Journal of Molecular Sciences, 21, 2074. 32197346 10.3390/ijms21062074 PMC7139911

[phy215977-bib-0015] Polgár, L. (2005). The catalytic triad of serine peptidases. Cellular and Molecular Life Sciences, 62, 2161–2172. 16003488 10.1007/s00018-005-5160-x PMC11139141

[phy215977-bib-0016] Rosato, S. , Unger, S. , Campos‐Xavier, B. , Caraffi, S. G. , Beltrami, L. , Pollazzon, M. , Ivanovski, I. , Castori, M. , Bonasoni, M. P. , Comitini, G. , Nikkels, P. G. J. , Lindstrom, K. , Umandap, C. , Superti‐Furga, A. , & Garavelli, L. (2022). Clinical and molecular diagnosis of Osteocraniostenosis in fetuses and newborns: Prenatal ultrasound, clinical, radiological and pathological features. Genes (Basel), 13, 261. 35205306 10.3390/genes13020261 PMC8871755

[phy215977-bib-0017] Sadideen, H. , & Swaminathan, R. (2004). Effect of acute oral calcium load on serum PTH and bone resorption in young healthy subjects: An overnight study. European Journal of Clinical Nutrition, 58, 1661–1665. 15305177 10.1038/sj.ejcn.1602026

[phy215977-bib-0018] Sato, T. , Courbebaisse, M. , Ide, N. , Fan, Y. , Hanai, J. I. , Kaludjerovic, J. , Densmore, M. J. , Yuan, Q. , Toka, H. R. , Pollak, M. R. , Hou, J. , & Lanske, B. (2017). Parathyroid hormone controls paracellular Ca(2+) transport in the thick ascending limb by regulating the tight‐junction protein Claudin14. Proceedings of the National Academy of Sciences of the United States of America, 114, E3344–e3353. 28373577 10.1073/pnas.1616733114 PMC5402431

[phy215977-bib-0019] Schigt, H. , Bald, M. , van der Eerden, B. C. J. , Gal, L. , Ilenwabor, B. P. , Konrad, M. , Levine, M. A. , Li, D. , Mache, C. J. , Mackin, S. , Perry, C. , Rios, F. J. , Schlingmann, K. P. , Storey, B. , Trapp, C. M. , Verkerk, A. J. M. H. , Zillikens, M. C. , Touyz, R. M. , Hoorn, E. J. , … de Baaij, J. H. F. (2023). Expanding the phenotypic Spectrum of Kenny‐Caffey syndrome. The Journal of Clinical Endocrinology and Metabolism, 108, e754–e768. 36916904 10.1210/clinem/dgad147 PMC10438882

[phy215977-bib-0020] Tan, R. S. G. , Lee, C. H. L. , Dimke, H. , & Todd Alexander, R. (2021). The role of calcium‐sensing receptor signaling in regulating transepithelial calcium transport. Experimental Biology and Medicine (Maywood, N.J.), 246, 2407–2419. 10.1177/15353702211010415 PMC8606958 33926258

[phy215977-bib-0021] Unger, S. , Górna, M. W. , Le Béchec, A. , Do Vale‐Pereira, S. , Bedeschi, M. F. , Geiberger, S. , Grigelioniene, G. , Horemuzova, E. , Lalatta, F. , Lausch, E. , Magnani, C. , Nampoothiri, S. , Nishimura, G. , Petrella, D. , Rojas‐Ringeling, F. , Utsunomiya, A. , Zabel, B. , Pradervand, S. , Harshman, K. , … Superti‐Furga, A. (2013). FAM111A mutations result in hypoparathyroidism and impaired skeletal development. American Journal of Human Genetics, 92, 990–995. 23684011 10.1016/j.ajhg.2013.04.020 PMC3675238

[phy215977-bib-0022] Wang, H. , Yang, H. , Shivalila, C. S. , Dawlaty, M. M. , Cheng, A. W. , Zhang, F. , & Jaenisch, R. (2013). One‐step generation of mice carrying mutations in multiple genes by CRISPR/Cas‐mediated genome engineering. Cell, 153, 910–918. 23643243 10.1016/j.cell.2013.04.025 PMC3969854

